# Progress in Anæsthetics

**Published:** 1899-07-22

**Authors:** 


### PROGRESS IN ANÆSTHETICS.

IT has been the prevailing opinion amongst anæsthetists and surgeons that nitrous oxide gas is the safest anæsthetic, after that ether, and, lastly, chloroform. The position assigned to ether has, however, been recently disputed, Dr. Whitney asserting that if the deaths from pneumonia after anæsthetisation be added to Guilt's statistics, the mortality from ether is far greater than that from chloroform; in fact, fully double.[1] Hankel says that it is a question by no means settled whether ether or chloroform is the more dangerous drug, whilst Müller asserts that the dangers of ether are at least as great as those of chloroform. The percentage of deaths from pneumonia following the administration of ether are variously stated by different observers, but they appear agreed that it follows most frequently after gynæcological operations; and, according to Dr. McCandie, especially those on parts in the abdomen above the level of the umbilicus. In contradiction of the above opinion, Mr. E. F. White asserts that ether is fully five times more reliable than chloroform, and remarks that we hear a great deal about cases having died from lung trouble following etherisation, but no mention is made of the number saved by its stimulating effect.[2]

That nitrous oxide gas is a true anæsthetic, and not only an asphyxiant, is now proved; further, that its administration mixed with air or oxygen does not in any way interfere with its anæsthetic properties. Working upon this principle Dr. Flux has introduced an original method of administering it by means of an open inhaler which is fitted accurately to the face but is widely open at the top. The gas is allowed to flow into the mouth of the inhaler, "it being only necessary to admit gas during the act of inspiration." Dr. Flux affirms that when anæsthesia has been once induced it can be kept up indefinitely by allowing the patient an occasional breath of gas, the inhaler being removed or not in the intervals between the breaths as may be convenient. It is also claimed for this method that it is unattended with any excitement, stertor, lividity, or convulsive movement, or any sign of asphyxia, the whole proceeding being characterised by extreme placidity. In criticising this method Mr. Bellamy Gardner maintains that there must be present in the inhaler a smaller percentage of air than is assumed by Dr. Flux. In experiments with Dr. Hewitt, Mr. Gardner found that with mixtures of nitrous oxide gas and air, the air being in the proportion of 30 per cent., true anæsthesia was almost unobtainable. He (Mr. Gardner) thinks that by reason of the aqueous vapour present in expiration, or some other physical reason, the expirations do not escape from Dr. Flux's inhaler to the degree one would expect. Dr. Flux has tried his method in a considerable number of cases, and all who have witnessed the administrations are agreed as to the ease with which the patient inhales the gas and his deeply placid condition throughout. Dr. Flux mentions an experiment in which a mouse was placed in the bottom of a pot, open at the top, and into which a little gas had been put. The mouse remained five and a half hours without moving a limb, but as soon as it was taken out it prepared to run away. Dr. Flux pricked the mouse in the interval frequently, but it apparently did not feel, because it did not move.

On the administration of nitrous oxide gas admixed with air or oxygen, Dr. Hewitt has made some original and valuable researches, the results of which he communicated in a paper read before the Royal Medical and Chirurgical Society. Some of the conclusions Dr. Hewitt arrived at were as follows[3]: That the asphyxial phenomena of pure nitrous oxide might be eliminated without interfering with the anæsthetic effects of the gas by administering with it certain proportions of oxygen, either pure or as atmospheric air. When the percentage of air or oxygen was considerable (30 per cent. of air or 13 per cent, of oxygen) respiration became noiseless and free from all obstruction. The most marked cynosis was met with with very small percentages of air (3 to 6 per cent.) or oxygen (under 3 per cent.). Cyanosis diminished as the percentages were increased, until with 30 per cent. of air it was very slight, whilst with 11 per cent. of oxygen it disappeared. Anoxæmic convulsions ceased to occur with mixtures containing 30 per cent. of air or 6 per cent. of oxygen. Reflex and excitement movements were most apparent with small percentages of air, or oxygen in considerable percentages, but were least likely to assert themselves with mixtures containing moderate quantities (from 12 to 16 per cent.) of air, or (from 3 to 7 per cent.) of oxygen. Phonated sounds were most likely to occur with nitrous oxide administered with large percentages of air or oxygen, less likely with small percentages, and least with moderate percentages, such as 6 to 10 per cent. air or 3 to 11 per cent. oxygen. The longest duration of available anæsthesia was obtained with mixtures containing 3 to 11 per cent. of oxygen, the maximum being with 7 per cent. The shortest available anæsthesia was recorded with nitrous oxide alone, and with nitrous oxide mixtures containing 30 per cent. of air. In estimating the respective merits of the various mixtures on anæsthetic agents, the best results were obtained with mixtures of nitrous oxide and oxygen, the worst with nitrous oxide alone. The results with nitrous oxide and air were more uncertain than those with nitrous oxide and oxygen. It was found that there was no one mixture of nitrous oxide with air or with oxygen which would successfully anæsthetise every patient. The best method of administering the mixture of nitrous oxide and oxygen was by means of a regulating apparatus by which the percentage of oxygen could be progressively increased from 2 or 3 per cent. to 7, 8, 9 or 10 per cent., the longer the administration lasted the greater might be the percentage of oxygen. For anæsthetising with mixtures of nitrous oxide and air it was found advisable to admit 14 to 18 per cent. of air for men and from 18 to 22 per cent. for women and children.

There appears to be some danger in administering chloroform after having commenced with a mixture of nitrous oxide gas and oxygen.[4] The latter mixture dilates the heart, and the chloroform, though in small quantity, may further dilate it until finally it inhibits it.

Professor Ramsay,[5] in a paper on "Pure Anæsthetics" read before the Society of Anæsthetists, called attention to the changes which take place in chloroform when administered in a gas-lit room, and also when kept for some time in a partially-filled bottle and exposed to sunlight. In the former case carbonyl chloride (phosgene, $C\,O\,Cl_2$) is evolved, giving rise to an acrid, pungent, and irritating odour. The equation showing this change is $C\,H\,Cl_3 + O = C\,O\,Cl_2 + H\,Cl$. Besides this the chloroform burns, and the oxygen of the air unites with the carbon to form carbonic acid, while the chlorine is liberated. To prevent this decomposition the makers add alcohol, as carbonyl chloride cannot exist in the presence of alcohol. Professor Ramsay thinks that some change takes place even in the presence of the alcohol, but it is a very slow one, and does not proceed to the same extent. Carbonyl chloride is at once destroyed by contact with an alkali; therefore, to keep chloroform sweet the addition of half-a-thimbleful of slaked lime is sufficient for a large quantity. Ether also, on keeping, acquires a sharp smell, and when breathed produces uncomfortable effects. On evaporation there is left a sticky residue giving the reactions of peroxide of hydrogen. The change is therefore probably due to the formation of ethyl peroxide. To keep ether pure the addition of a thimbleful of mercury; the bottle being occasionally well shaken, is all that is necessary. The mercury becomes coated with a black powder. To purify the A. C. E. mixture it is necessary to add both slaked lime and mercury. Professor Ramsay also gave the results of experiments relating to the evaporation of the gases from a mixture of ether and chloroform, made in proportion to their molecular weights. It was shown that at the end of the experiment ether remained with the chloroform in fair quantity. "Thus when 100 per cent. of the mixture had been evaporated the percentage of chloroform in the vapour was 62."[6] Professor Ramsay considers that the presence of alcohol in the A. C. E. mixture only serves to diminish the proportion of ether and chloroform in the total vapour.

Dr. W. J. Mèlardie,[6] in a summary under the heading of Status Thymicus and Chloroform Narcosis, remarks that Gruwitz, of Grefowald, noted the occurrence of an enlargement of the thymus gland in two cases of death under chloroform, and emphasised the possibility of a connection between such enlargement and the fatal results. Paltauf collected a large number of cases of sudden deaths in adults in which post-mortem examination gave great weight to the correctness of the above theory. Enlargement was found in the tonsils, lymph follicles, lymph gland system, follicles at the base of the tongue, the spleen, and lastly, the thymus gland, which varied in different subjects; there was also in most cases some narrowing of the aorta. This condition was described as the "Lymphatico-chlorotic constitution." The cause of sudden death was attributed to cardiac paralysis, and not to mechanical interference by pressure. Other cases have also been recorded by Kendrat. In records of deaths under ether no mention has been made of this peculiar condition; it is therefore inferred that chloroform in those suffering from this diathesis is particularly dangerous, and in young anæmic people with enlarged tonsils and follicles at the base of the tongue, generally swollen glands, enlarged spleen and increase of the heart dulness upwards, chloroform should be avoided.

[1] Treatment, Jan. 26, 1899. [2] Clin. Jour., April 19, 1899. [3] Lancet, Feb. 18, 1899. [4] Lancet, April 22, 1899. [5] Clin. Jour., Dec. 7, 1898. [6] Treatment, Jan. 26, 1899.

